# Mesenchymal stem cell-derived exosomal miR-26a induces ferroptosis, suppresses hepatic stellate cell activation, and ameliorates liver fibrosis by modulating SLC7A11

**DOI:** 10.1515/med-2024-0945

**Published:** 2024-05-13

**Authors:** Ying Cao, Huan Yang, Yan Huang, Jian Lu, Hong Du, Bingying Wang

**Affiliations:** Department of Clinical Laboratory, The Second Affiliated Hospital of Soochow University, Suzhou, Jiangsu, China

**Keywords:** fibrosis, mesenchymal stem cell, exosome, miR-26a, ferroptosis, SLC7A11

## Abstract

Liver fibrosis is a key contributor to hepatic disease-related mortality. Exosomes derived from mesenchymal stem cells (MSCs) have been revealed to improve liver fibrosis. To explore the effect and mechanism of MSC-derived exosomal miR-26a on liver fibrosis, exosomes were separated from bone marrow-derived MSCs (BMSCs) and used to treat with LX2 cells. The miR-26a level was decreased in BMSC-derived exosomes. Treatment with exosomes isolated from human BMSCs transfected with miR-26a mimics (miR-26a mimic-Exo) decreased the 5-ethynyl-2'-deoxyuridine-positive cell rate, the protein level of α-SMA and collagen I, and the glutathione (GSH) level but enhanced the apoptosis rate and the reactive oxide species (ROS) level in LX2 cells, which were reversed by the treatment of deferoxamine. Mechanically, miR-26a directly bound SLC7A11 mRNA and negatively modulated the level of SLC7A11 in LX2 cells. Overexpression of SLC7A11 reversed the miR-26a mimic-Exo-induced alterations in the level of ROS, Fe^2+^, malonaldehyde, and GSH in LX2 cells. *In vivo*, miR-26a mimic-Exo decreased the level of SLC7A11 and attenuated CCL4-induced liver fibrosis. Collectively, miR-26a mimic-Exo induced ferroptosis to alleviate liver fibrosis by regulating SLC7A11, which may provide new strategies for the treatment of liver fibrosis, and even other relevant diseases.

## 1 Introduction

Liver fibrosis is a wound-healing progress in response to chronic hepatic damage, which can be caused by a variety of factors, such as alcohol, hepatitis viruses, diabetes, obesity, and hepatotoxic drugs [1]. Liver fibrosis, characterized by an excessive increase in the extracellular matrix (ECM) proteins in hepatic stellate cells (HSCs), can progress to cirrhosis and even ultimately result in hepatic failure and hepatocellular carcinoma and is one of the crucial causes of incidence rate and death rate around the world [1,2,3]. Experimental and clinical evidence indicate that the pathological conditions of liver fibrosis can be reversible [1,3]. However, no effective drugs are currently approved to treat liver fibrosis. Some compounds have been reported to exhibit antifibrotic properties, but the inadequate efficacy with various side effects greatly impedes their clinical application [4]. Thus, identifying the target and developing the strategies based on the pathogenesis is of great significance for the treatment of liver fibrosis.

Induction of programmed cell death of activated HSCs, such as apoptosis, ferroptosis, and necroptosis, can improve the pathological progression of liver fibrosis [5,6]. Ferroptosis is an iron-dependent lipid peroxidation that differs from apoptosis, pyroptosis, or necrosis [7,8]. Ferroptosis is closely related to the progression of multifarious liver disorders. For instance, Zhang et al. [9] report that BRD7-P53-SLC25A28 axis modulates ferroptosis in HSCs. The study from the same research group showed that the RNA-binding protein ZFP36/TTP regulates autophagy to prevent ferroptosis in HSCs [10]. Artemether has been identified as a potential medicine for the therapy of liver fibrosis, in which artemether promotes the extensive production of reactive oxide species (ROS) and the occurrence of ferroptosis by inhibiting the ubiquitination of iron regulatory protein 2 to cause its deposition and the enhancement of iron in HSCs [11]. Tsurusaki and colleagues revealed that hepatic ferroptosis triggers the initiation of inflammation in nonalcoholic steatohepatitis [12]. Metallothionein-1G suppresses ferroptosis to accelerate sorafenib resistance in hepatocellular carcinoma cells [13]. Therefore, treating liver fibrosis by inducing ferroptosis in activated HSCs may be a promising approach [10,14].

Exosomes with a diameter of 30–100 nm are bilayer lipid vesicles that have been proven to have no risk for tumor formation and have low immunogenicity [15]. Exosomes can be actively secreted into the extracellular circumstances by diverse mammalian cells [16], and mesenchymal stem cell (MSC)-derived exosomes have been revealed to improve liver fibrosis [17,18]. Exosomes carry a large number of bioactive molecules, such as lipids, proteins, nucleic acids, non-coding ribonucleic acid (RNA), which can be functionally exchanged between cell types and even across species [19]. microRNA (miRNA), a type of noncoding RNAs with 21–25 nucleotides, can conjugate with RNA-elicited silencing complex to bind on the 3′-untranslated region of target mRNA, which then restrains the formation of the translation initiation complex, thereby leading to the translation inhibition [20]. Therefore, miRNAs play a crucial role in various human diseases, including in liver fibrosis [21]. miR-26 has exhibited the inhibitory effect on myocardial fibrosis [22,23], kidney fibrosis [24], and pulmonary fibrosis [25]. It has been reported that miR-26a ameliorates ethanol-induced acute liver injury by enhancing autophagy [26]. miR-26a is specifically induced by endoplasmic reticulum stress and is involved in the progression of nonalcoholic fatty liver disease [27]. miR-26a also directly participates in the liver regeneration by targeting the mdm2/p53 loop [28]. Notably, miR-26a is differentially expressed between HSCs and liver tissues, suggesting that miR-26a is related to liver fibrosis [29]. However, the specific role and mechanism of miR-26a in liver fibrosis remains unclear.

This study aimed to explore the effect and potential mechanism of MSC-derived exosomal miR-26a on liver fibrosis. The findings in this study will help to further understand the role of MSC-derived exosomal miR-26a in the pathogenesis of liver fibrosis and may provide a new approach for the therapy of liver fibrosis, and other relevant disorders.

## 2 Materials and methods

### 2.1 Cell culture

Human bone marrow-derived mesenchymal stem cells (BMSCs) were bought from the American Type Culture Collection (Cat#: PCS-500-012, ATCC, Manassas, VA, USA) and grown in Mesenchymal Stem Cell Basal Medium (Cat#: PCS-500-030, ATCC) accompanied with Mesenchymal Stem Cell Growth Kit (Cat#: PCS-500-041, ATCC) in a 37°C and 5% CO_2_ incubator. Human HSC LX2 cells were obtained from Procell (Wuhan, China) and cultured in DMEM (Cat#: PM150210, Procell) added with 10% fetal bovine serum (FBS, Cat#: 12103C, Merck, Whitehouse Station, NJ, USA) and 1% penicillin/streptomycin (P/S, Cat#: PB180120, Procell) with 5% CO_2_ at 37°C.

### 2.2 Cell transfection

miR-26a-mimic and relevant negative control mimic (NC-mimic) were prepared by GenePharma (Shanghai, China). The SLC7A11 sequences were inserted into pcDNA vector plasmids (designated as SLC7A11) to overexpress SLC7A11, with empty pcDNA as NC (Vector). The miR-26a-mimic, NC-mimic, SLC7A11, and Vector were infected into BMSCs by Lipofectamine™ Stem Transfection Reagent (Cat#: STEM00001, Invitrogen, Carlsbad, CA, USA) based on the operation instruction. Besides, the miR-26a-mimic and NC-mimic were infected into LX2 cells through Lipofectamine 3000 (Cat#: L3000001, Invitrogen) in keeping with the operating manual.

### 2.3 Isolation and identification BMSC-derived exosomes

The exosomes were isolated from the culture medium of BMSCs using an exosome isolation reagent (Cat#: 4478359, Invitrogen). Briefly, the media of BMSCs were centrifuged at 2,000 × *g* for half an hour to harvest the supernatant. Then, the Total Exosome Isolation reagents were appended into the supernatant and vortexed for the sufficient mixture. The mixture was hatched at 4°C for 24 h and centrifuged at 10,000 × *g* for 60 min to collect the pellet. The pellet was re-suspended into phosphate buffer saline (PBS, Cat#: P1020, Solarbio, Beijing, China) to obtain the isolated BMSC-derived exosomes (BMSC-Exo). The BMSC-Exo was imaged under transmission electron microscopy (TEM) (Cat#: 1200EX, Jeol, Japan). Besides, the exosomal markers, containing CD9, CD63, and calnexin, were examined via western blot after the treatment of the RIPA solution (Cat#: R0010, Solarbio).

### 2.4 LX2 cells’ treatment

LX2 cells were plated into the six-well plates with a density of 5  ×  10^5^ cells/well and incubated in exosome-exhausted culture medium with 10% FBS and 1% P/S. Then, 50 μg/mL exosomes separated from transfected MSCs were used to incubate with the LX2 cells. Besides, to further verify the effect of BMSC-derived exosomal miR-26a on ferroptosis, LX2 cells were administrated with miR-26a mimic-Exo as well as the ferroptosis inhibitor, deferoxamine (DFO, 100 µM) for 24 h.

### 2.5 The 5-ethynyl-2′-deoxyuridine (EdU) incorporation experiment

LX2 cells were treated with the BeyoClick™ EdU Cell Proliferation Kit with Alexa Fluor 647 (Cat#: C0081S, Beyotime, Shanghai, China) for the determination of proliferation according to the instruction for use. Hoechst 33342 (5 μg/mL, Cat#: C0031, Solarbio) was employed to re-stain the cell nucleus. The pictures were photographed by a fluorescence microscopy (Olympus, Tokyo, Japan).

### 2.6 Flow cytometry

As the previous studies [30,31], cell apoptosis was analyzed by flow cytometry with an Annexin V-FITC Apoptosis Detection Kit (Cat#: CA1020, Solarbio). Cells were rinsed with pre-cold PBS and mixed into binding buffer. Cells were dyed with FITC-Annexin V and propidium iodide for 5 min in a dark. Cells were examined on a FACScan flow cytometry (BD Biosciences, NJ, USA) and the apoptotic rate was determined by BD CellQuest Pro software (version 5.1, BD Biosciences). In addition, the ROS level was measured using a reactive oxygen species assay kit (Cat#: CA1410, Solarbio) and analyzed via flow cytometry.

### 2.7 Evaluation of ferroptosis-related indicators

The level of Fe^2+^, malonaldehyde (MDA), and glutathione (GSH) was analyzed by using Iron Assay kit (Cat#: ab83366, Abcam, Cambridge, UK), Lipid Peroxidation (MDA) Assay kit (Cat#: ab118970, Abcam), and GSH Assay kit (Cat#: ab239727, Abcam) based on the operating manual.

### 2.8 Dual-luciferase reporter assay

The binding sites between miR-26a and SLC7A11 mRNA were predicted by the miRDB and targetScan online website. LX2 cells were seeded into the 24‑well plates and grown with 5% CO_2_ at 37°C. When the cells’ confluency reached 70–80%, the transfection was conducted. Wild-type SLC7A11 (SLC7A11-WT) and mutant SLC7A11 (SLC7A11-MUT) were sub-cloned into pGL3-Basic luciferase vector (2 μg/mL, GenePharma) and then transfected into LX2 cells with miR-26a-mimic or NC-mimic using Lipofectamine™ 3000 (Cat#: L3000001, Invitrogen). Forty-eight hours later, luciferase activity was measured by a Dual Luciferase Reporter Gene Assay Kit (Cat#: RG027, Beyotime) based on the instruction for use.

### 2.9 *In vivo* experiments

Four-week-old male C57BL/6J mice (13–15 g) were acquired from Junke biological Co., LTD. (Nanjing, China) and fed in the laboratory environment with 12 h/12 h light–dark cycle and 40–60% the relative humidity at 22℃. All animal experiments were abided by the Guide for the Care and Use of Laboratory Animals [32] and authorized by the Animal Research Ethics Committee of the Second Affiliated Hospital of Soochow University. The liver fibrosis model was constructed through the application of CCl4 (Cat#: 488488, Sigma-Aldrich, St. Louis, MO, USA) dissolved in olive oil with a ratio of 1:10 based on the previous description [33]. Mice were randomized into four groups, including control, CCl4, CCl4 + NC mimic-Exo, and CCl4 + miR-26a mimic-Exo. Mice in CCl4, CCl4 + NC mimic-Exo, and CCl4 + miR-26a mimic-Exo were modeled by intraperitoneally injecting with 0.5 mg/kg CCl4 for 8 weeks twice a week. Twenty-four hours after the model, mice were administrated with PBS, exosomes separated from MSCs infected with NC mimic (100 μg/mL), and exosomes from MSCs with miR-26a mimic (100 μg/mL) through the tail vein, respectively. The dose of exosomes applied in the present study was based on the previous research [34]. Mice were sacrificed by inhaling the excess isoflurane (Cat#: R510-22, RWD, Guangdong, China), and the blood and liver tissues were yielded for the following experiments.

### 2.10 Masson staining

Liver tissues were collected and fixed in 4% paraformaldehyde (Cat#: P1110, Solarbio). Fixed tissues were embedded into paraffin (Cat#: YA0011, Solarbio) and cut into sections (5 μm). Sections were stained with the Masson’s Trichrome Stain kit (Cat#: G1340, Solarbio) for the determination of the degree of liver fibrosis. Pictures were obtained under a light microscope (Olympus).

### 2.11 Measurement of aminotransferase (ALT) and aspartate aminotransferase (AST)

Blood was collected and stood for 2 h at room temperature, and at 4°C overnight. Subsequently, blood samples were centrifuged at 4,000 × *g* for 10 min at 4°C to isolate the serum samples. The serum contents of ALT and AST were detected by using Alanine Transaminase Activity Assay kit (Cat#: ab105134, Abcam) and Aspartate Aminotransferase Activity Assay kit (Cat#: ab105135, Abcam) according to the operation instruction.

### 2.12 Reverse transcription (RT) reaction and quantitative real-time polymerase chain reaction (RT-qPCR)

Based on the pervious study [35], total RNA was obtained from cells and tissues through TRIzol reagent (Cat#: 15596026, Invitrogen). The RT reaction was conducted with the Bulge-Loop™ miRNA RT-qPCR Primer (Cat#: C10211, RiboBio Co., Ltd., Guangzhou, China) at 42°C for 60 min and at 70°C for 10 min. RT-qPCR was executed on the Bio-Rad CFX Manager software (Bio-Rad Laboratories, Inc.) with 2× SYBR Master mix (Cat#: RR820A, Takara, Dalian, China). The miR-26a level was tested at 94°C for 10 min, followed by 40 cycles at 94°C for 5 s, 58°C for 15 s, and 70°C for 20 s. The miR-26a expression was analyzed by the 2^−ΔΔCT^ method normalized with U6. The primer sequences were: 5′-TCCGTTGTTTCAAGTAATCCAGG-3′ (miR-26a upstream), 5′-ATCAACCACACGTCATGTGACT-3′ (miR-26a downstream), 5′-CTCGCTTCGGCAGCACA-3′ (U6 upstream), and 5′-AACGCTTCACGAATTTGCGT-3′ (U6 downstream).

### 2.13 Western blot

As the previous descriptions [36,37], cells and tissues were treated with RIPA lysis buffer to extract the total proteins and then quantified with a BCA kit (Cat#: PC0020, Solarbio). 20 μg proteins were electrophoresed with 10% sodium dodecyl sulfate-polyacrylamide gel electrophoresis and transferred onto polyvinylidene fluoride membranes (Cat#: IPVH00010, EMD Millipore, Billerica, MA, USA) for the routine manipulation of western blot experiments. After the membranes were developed with ECL reagents (Cat#: SW2030, Solarbio), the band intensity was determined by ImageJ software (National Institutes of Health, USA). The primary antibodies included anti-CD9 (Cat#: ab223052, 1:1,000), anti-CD63 (Cat#: ab134045, 1:5,000), anti-Calnexin (Cat#: ab22595, 1:1,000), anti-α-SMA (Cat#: ab5694, 1:1,000), anti-collagen I (Cat#: ab34710, 1:5,000), anti-SLC7A11 (Cat#: ab216876, 1:1,000), and anti-β-actin (1:1,000, Cat#: ab8227). The secondary antibodies were goat anti-rabbit IgG H&L (HRP) (Cat#: ab6721, 1:10,000). All the antibodies were from Abcam.

### 2.14 Statistical analysis

Data were displayed as mean ± standard deviation. The statistical difference was determined via the Student’s *t*-test and one-way analysis of variance for two groups and more than two groups followed by *post hoc* Bonferroni test by SPSS 26.0 software (IBM, Armonk, New York, USA). *p* < 0.05 was defined as the significant difference.


**Ethics approval:** Ethical approval was obtained from the Ethics Committee of the Second Affiliated Hospital of Soochow University.

## 3 Results

### 3.1 miR-26a was lowly expressed in BMSC-derived exosomes

**Figure 1:**
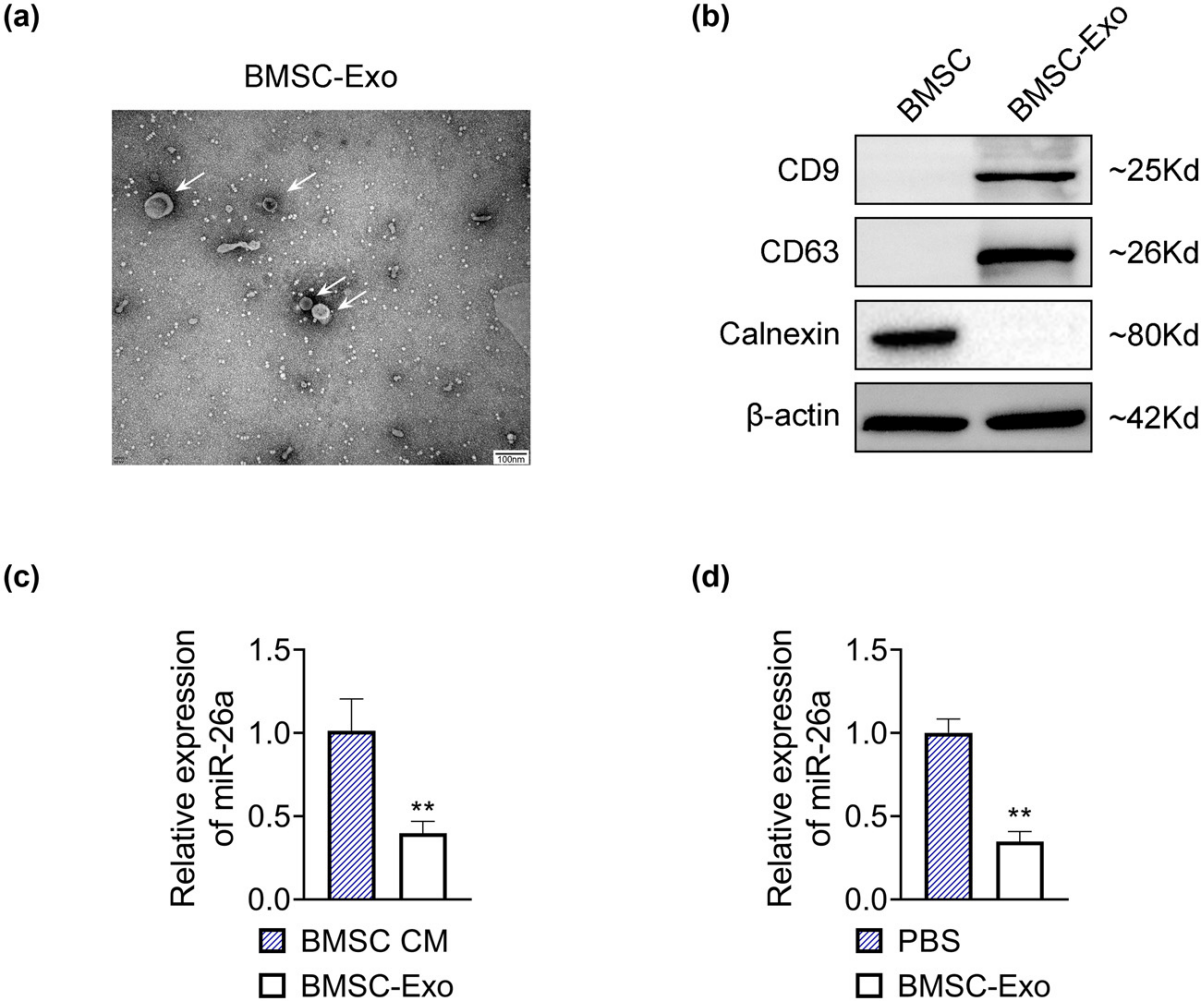
miR-26a was lowly expressed in BMSC-derived exosomes. (a) The exosomes were isolated from human BMSCs and then observed by TEM. Scale bar = 100 nm. The exosomes were labeled with white arrows. (b) The relative protein expression of CD9, CD63, and calnexin was examined by western blot. Data were presented after being normalized with β-actin. (c) The relative expression of miR-26a in BMSC-Exo or BMSC-CM was detected by RT-qPCR. Data were presented after being normalized with *U6*. ^**^ *p* < 0.05 vs BMSC-CM. (d) The relative expression of miR-26a in LX2 cells treated with BMSC-Exo or PBS was detected by RT-qPCR. Data were presented after being normalized with *U6*. ^**^ *p* < 0.05 vs PBS.

To explore the effect of MSC-derived exosomal miR-26a on liver fibrosis, the exosomes were isolated from human BMSCs. Small round or oval vesicles with uneven morphology were observed under TEM, with a diameter of 30–100 nm (Figure 1a), morphologically confirming the successful isolation of the exosomes. In addition, both CD9 and CD63 were expressed in BMSC-Exo, while calnexin was not expressed in BMSC-Exo (Figure 1b), further verifying the exosomes in molecular level. These results indicated that exosomes were successfully isolated from human BMSCs. Then, the expression level of miR-26a was examined in exosomes. As shown in Figure 1c, the miR-26a level was markedly decreased in BMSC-Exo relative to that in BMSC cellular matrix (BMSC-CM). Moreover, the miR-26a expression in LX2 cells administrated with BMSC-Exo was significantly reduced compared with LX2 cells treated with PBS (Figure 1d). Together, these results suggested that the miR-26a level was reduced in BMSC-derived exosomes.

### 3.2 Overexpression of BMSC-derived exosomal miR-26a inhibited the HSC activation

**Figure 2:**
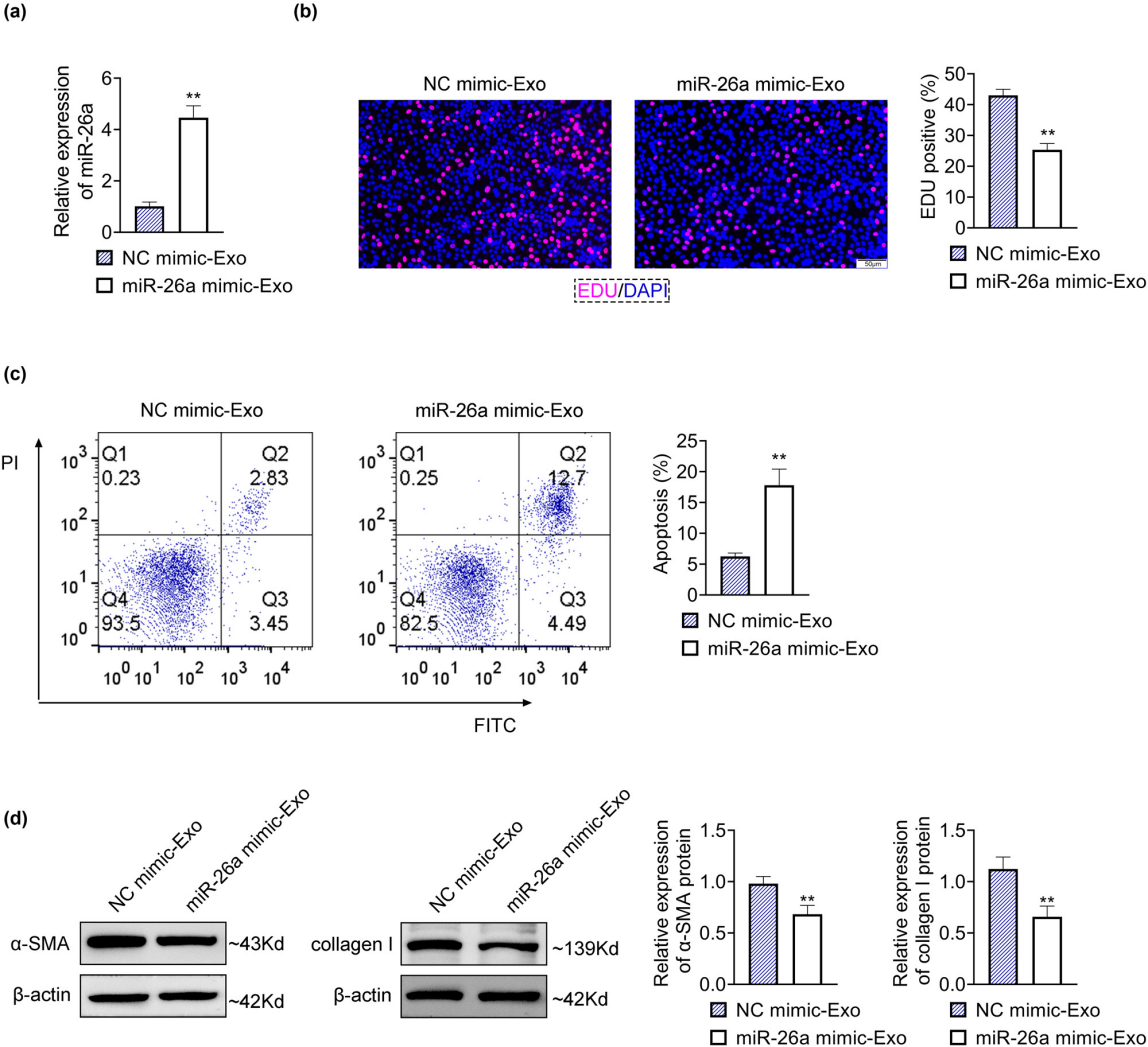
Overexpression of BMSC-derived exosomal miR-26a inhibited the HSC activation. (a) miR-26a mimics were transfected into human BMSCs to upregulate the level of miR-26a, and then, the exosomes were isolated from human BMSCs. The relative expression of miR-26a in miR-26a mimic-Exo or NC mimic-Exo was examined by RT-qPCR. Data were presented after being normalized with *U6*. (b) The proliferation of LX2 cells treated with miR-26a mimic-Exo or NC mimic-Exo was assessed by EDU assays. Scale bar = 50 nm. (c) The apoptosis rate of LX2 cells treated with miR-26a mimic-Exo or NC mimic-Exo was measured by flow cytometry. (d) The relative protein expression of α-SMA and collagen I of LX2 cells treated with miR-26a mimic-Exo or NC mimic-Exo was detected by western blot. Data were presented after being normalized with β-actin. ^**^ *p* < 0.05 vs NC mimic-Exo.

HSC activation is a key pathogenesis of liver fibrosis; thus, the role of BMSC-derived exosomal miR-26a was assessed in the HSC activation. Since the expression level of miR-26a was decreased in BMSC-derived exosomes, miR-26a mimics were transfected into human BMSCs to upregulate the level of miR-26a, and then, the exosomes were isolated from human BMSCs. The miR-26a level was notably increased in exosomes isolated from human BMSCs transfected with miR-26a mimics (miR-26a mimic-Exo) compared with exosomes isolated from human BMSCs transfected with NC mimics (NC mimic-Exo) (Figure 2a), demonstrating a viable transfection efficiency. Treatment of miR-26a mimic-Exo prominently diminished the EDU-positive rate but markedly enhanced the apoptosis rate of LX2 cells compared with LX2 cells treated with NC mimic-Exo (Figure 2b and c). ECM deposition that can be promoted by the activation of HSC is a key characteristic of liver fibrosis. The protein levels of α-SMA and collagen I, two important proteins in ECM deposition, were significantly reduced in LX2 cells treated with miR-26a mimic-Exo compared with LX2 cells treated with NC mimic-Exo (Figure 2d). Collectively, these results indicated that upregulation of BMSC-derived exosomal miR-26a repressed the HSC activation.

### 3.3 Upregulation of BMSC-derived exosomal miR-26a induced ferroptosis

**Figure 3:**
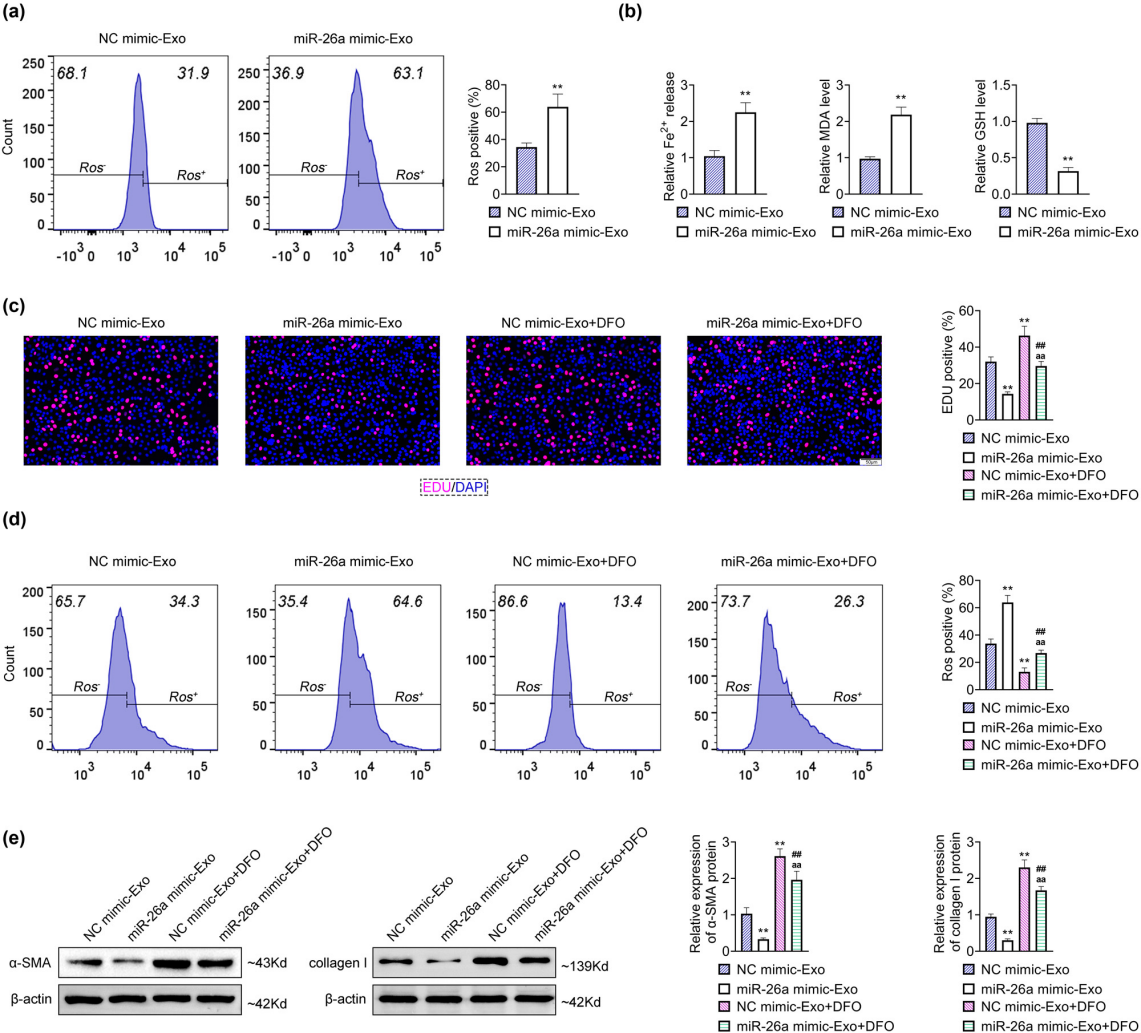
Overexpression of BMSC-derived exosomal miR-26a accelerated ferroptosis in HSCs. (a) The ROS level of LX2 cells treated with miR-26a mimic-Exo or NC mimic-Exo was detected by flow cytometry. (b) Measurement of Fe^2+^, MDA, and GSH of LX2 cells treated with miR-26a mimic-Exo or NC mimic-Exo. (c) The proliferation was assessed by EDU assays after LX2 cells were treated with miR-26a mimic-Exo, NC mimic-Exo, and DFO. Scale bar = 50 nm. (d) The ROS level was detected by flow cytometry after LX2 cells were treated with miR-26a mimic-Exo, NC mimic-Exo, and DFO. (e) The relative protein expression of α-SMA and collagen I was determined by western blot after LX2 cells were treated with miR-26a mimic-Exo, NC mimic-Exo, and DFO. Data were presented after being normalized with β-actin. ^**^ *p* < 0.05 vs NC mimic-Exo; ^##^ *p* < 0.05 vs miR-26a mimic-Exo; ^aa^ *p* < 0.05 vs NC mimic-Exo + DFO.

Then, the effect of BMSC-derived exosomal miR-26a on ferroptosis was detected. A prominent enhancement in the level of ROS, Fe^2+^, and MDA and a conspicuous decrease in the GSH level were observed in LX2 cells treated with miR-26a mimic-Exo compared with LX2 cells treated with NC mimic-Exo (Figure 3a and b), suggesting that BMSC-derived exosomal miR-26a induced ferroptosis in LX2 cells. To further verify the effect of BMSC-derived exosomal miR-26a on ferroptosis, LX2 cells were transfected with miR-26a mimic-Exo and treated with the ferroptosis inhibitor, DFO. Enhanced EDU-positive rate in LX2 cells treated with DFO was significantly counteracted by the incubation of miR-26a mimic-Exo, while reduced EDU-positive rate in LX2 cells treated with miR-26a mimic-Exo was markedly restored with the further treatment of DFO (Figure 3c). However, the opposite results were found in the ROS level (Figure 3d). Moreover, increased protein level of α-SMA and collagen I in LX2 cells with DFO were significantly counteracted with the incubation of miR-26a mimic-Exo, while decreased protein levels of α-SMA and collagen I in LX2 cells with miR-26a mimic-Exo were markedly restored with the further treatment of DFO (Figure 3e). Collectively, these results demonstrated that overexpression of BMSC-derived exosomal miR-26a promoted ferroptosis in HSCs.

### 3.4 MiR-26a targeted SLC7A11

**Figure 4:**
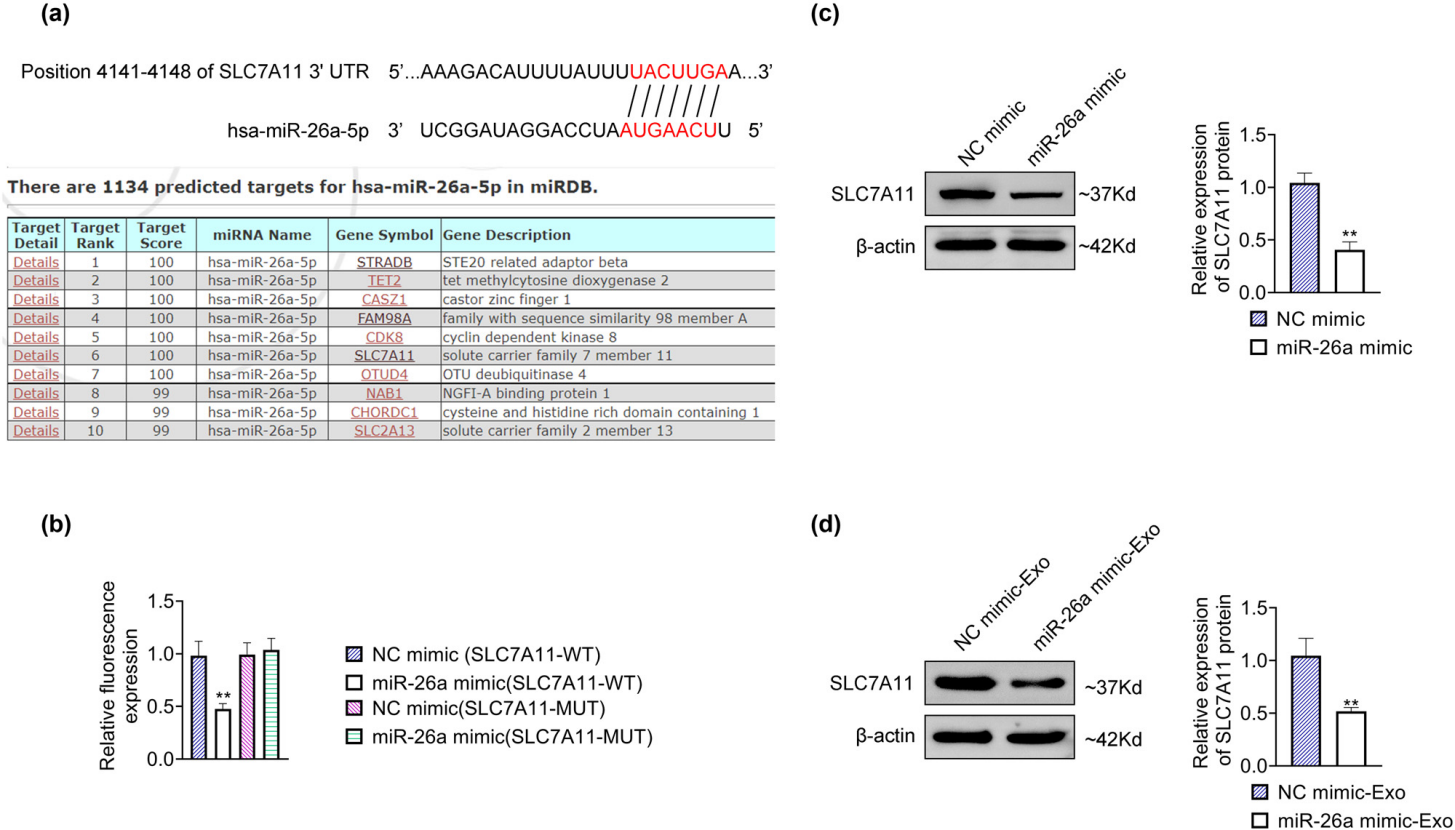
miR-26a directly targeted SLC7A11. (a) The binding sites between miR-26a and SLC7A11 mRNA were predicted, and the target rank and target score between miR-26a and SLC7A11 mRNA were shown. (b) The direct interaction between miR-26a and SLC7A11 was determined by a dual-luciferase reporter assay. (c) The relative protein expression of SLC7A11 was examined by western blot after LX2 cells were transfected with miR-26a mimic or NC mimic. Data were presented after being normalized with β-actin. (d) The relative protein expression of SLC7A11 was detected by western blot after LX2 cells were treated with miR-26a mimic-Exo. Data were presented after being normalized with β-actin. ^**^ *p* < 0.05 vs NC mimic-Exo.

To further explore the mechanism of miR-26a in liver fibrosis, the downstream targets of miR-26a were predicted by targetScan and miRDB. Binding sites were discovered between miR-26a and SLC7A11 mRNA due to the complementary base pairing, and the target rank and target score demonstrated the high possibility of the interaction between miR-26a and SLC7A11 mRNA (Figure 4a). Moreover, the relative fluorescence expression was markedly reduced in LX2 cells co-transfected with miR-26a mimic and SLC7A11-WT compared with LX2 cells transfected with NC mimic and SLC7A11-WT, while no difference was found in the relative fluorescence level in LX2 cells co-transfected with miR-26a mimic and SLC7A11-MUT, and cells co-transfected with NC mimic and SLC7A11-MUT (Figure 4b), suggesting that miR-26a could directly bind to SLC7A11. Transfection of miR-26a mimic into LX2 cells prominently decreased the relative protein level of SLC7A11 (Figure 4c), indicating that miR-26a negatively regulated the expression level of SLC7A11 in LX2 cells. Moreover, the SLC7A11 protein level was notably declined in LX2 cells treated with miR-26a mimic-Exo (Figure 4d). Altogether, these results expounded that miR-26a directly bound to SLC7A11 and negatively modulated the level of SLC7A11 in HSCs.

### 3.5 BMSC-derived exosomal miR-26a evoked ferroptosis of HSC by regulating SLC7A11

**Figure 5:**
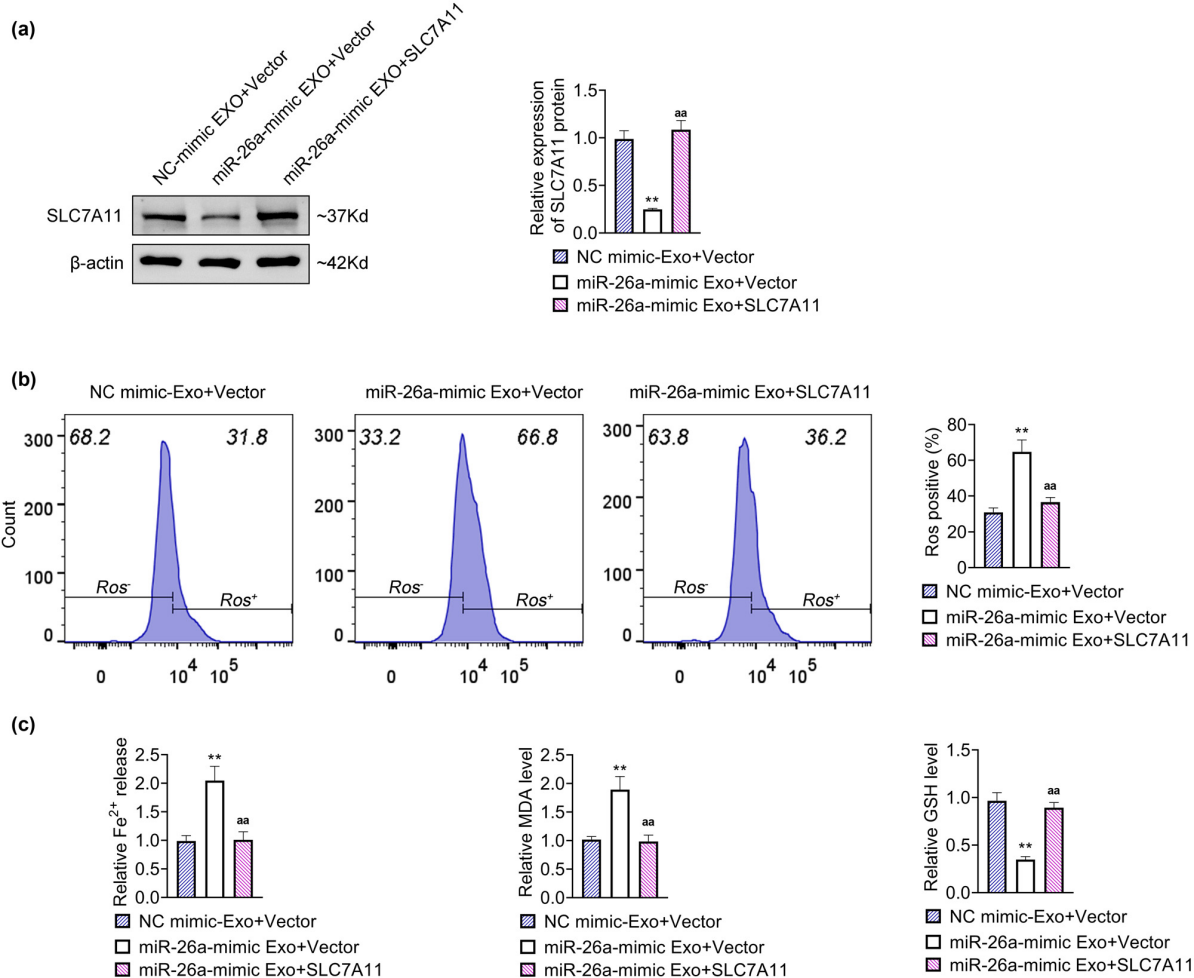
BMSC-derived exosomal miR-26a induced ferroptosis of HSC by regulating SLC7A11. LX2 cells were treated with miR-26a mimic-Exo as well as transfected with pcDNA vector plasmids carrying the sequences of SLC7A11. (a) The relative protein expression level of SLC7A11 was detected by western blot. Data were presented after being normalized with β-actin. (b) The ROS level was detected by flow cytometry. (c) Measurement of Fe^2+^, MDA, and GSH. ^**^ *p* < 0.05 vs NC mimic-Exo; ^aa^ *p* < 0.05 vs miR-26a mimic-Exo + Vector.

To investigate the direct role of SLC7A11 in BMSC-derived exosomal miR-26a-mediated ferroptosis, LX2 cells were administrated with miR-26a mimic-Exo and transfected with pcDNA vector plasmids carrying the sequences of SLC7A11 (designated as SLC7A11). The relative protein expression of SLC7A11 was significantly decreased with the treatment of miR-26a mimic-Exo, which was rescued with the overexpression of SLC7A11 in LX2 cells (Figure 5a). The ROS level was notably increased in LX2 cells treated with miR-26a mimic-Exo, which was markedly attenuated with the further overexpression of SLC7A11 (Figure 5b). Additionally, enhanced level of Fe^2+^ and MDA in LX2 cells treated with miR-26a mimic-Exo was prominently antagonized with the further overexpression of SLC7A11, while decreased level of GSH in LX2 cells treated with miR-26a mimic-Exo was observably rescued with the further overexpression of SLC7A11 (Figure 5c). Therefore, these results indicated that BMSC-derived exosomal miR-26a elicited ferroptosis of HSC by regulating SLC7A11.

### 3.6 BMSC-derived exosomal miR-26a alleviated CCL4-induced liver fibrosis *in vivo*

**Figure 6:**
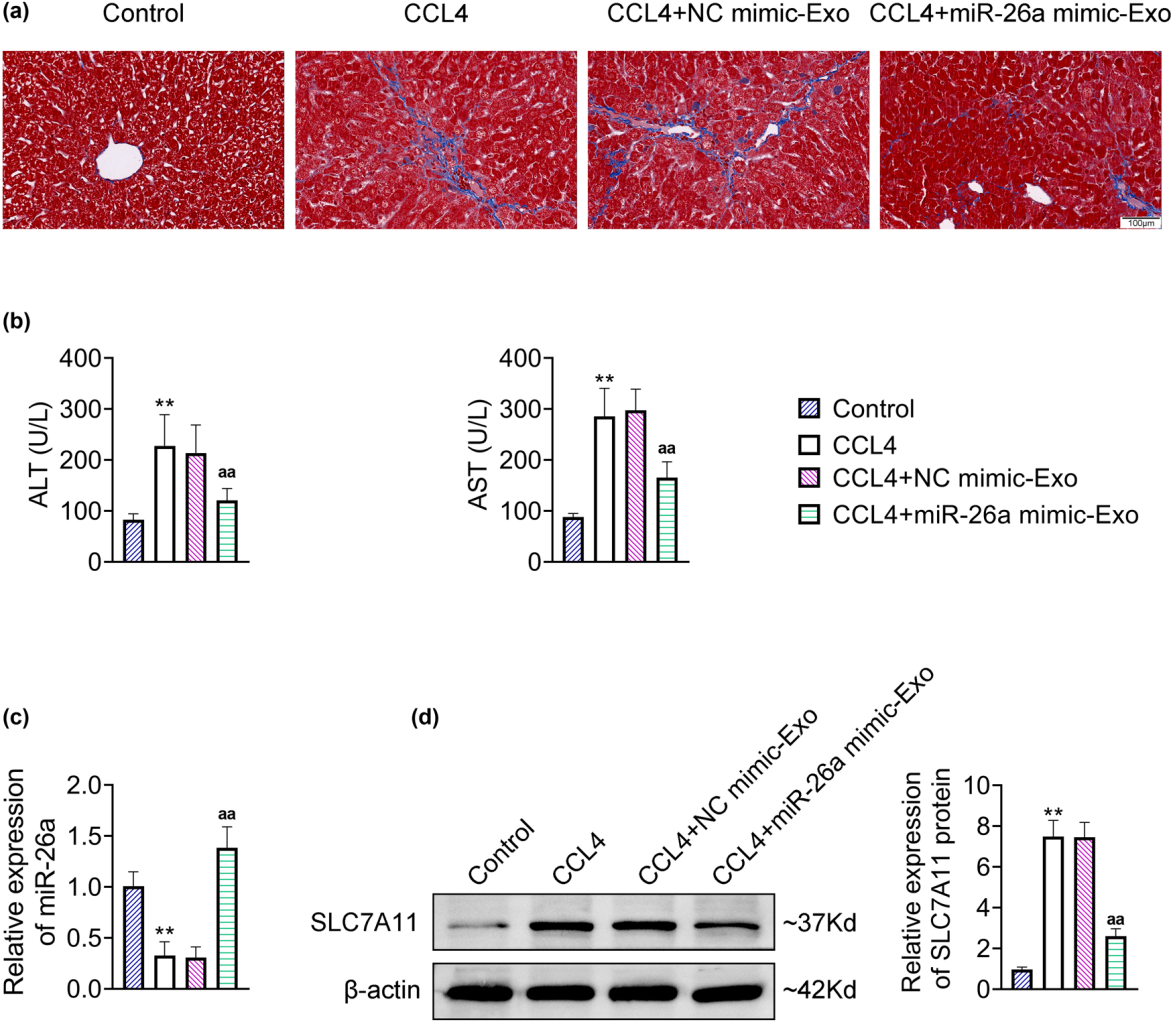
BMSC-derived exosomal miR-26a alleviated CCL4-induced liver fibrosis in mice. Mice were modeled by intraperitoneally injecting with 0.5 mg/kg CCl4 for 8 weeks twice a week. Twenty-four hours after the model, mice were injected with 100 μg/mL exosomes isolated from MSCs through the tail vein. (a) The liver fibrosis was assessed by Masson staining. (b) Examination of ALT and AST in sera. (c) The relative expression of miR-26a was detected by RT-qPCR. Data were presented after being normalized with *U6*. (d) The relative protein expression of SLC7A11 was detected by western blot. Data were presented after being normalized with β-actin. ^**^ *p* < 0.05 vs Control; ^aa^ *p* < 0.05 vs CCL4 + NC mimic-Exo.

Moreover, the effect of BMSC-derived exosomal miR-26a on liver fibrosis was evaluated in CCL4-induced mice, an *in vivo* model of liver fibrosis. Masson staining results showed that CCL4 evoked an obvious fibrosis in mice, which was profoundly improved by injecting of miR-26a mimic-Exo (Figure 6a). The serum contents of ALT and AST were significantly augmented in CCL4-induced mice, which were prominently declined with the injection of miR-26a mimic-Exo (Figure 6b). Low level of miR-26a in CCL4-induced mice was markedly restored with the injection of miR-26a mimic-Exo (Figure 6c). In addition, CCL4 triggered a prominent enhancement in the SLC7A11 protein level, which was observably neutralized by injecting of miR-26a mimic-Exo (Figure 6d). Together, these results indicated that BMSC-derived exosomal miR-26a reduced the level of SLC7A11 and relieved CCL4-induced liver fibrosis in mice.

## 4 Discussion

MSCs have attracted more and more attention for the treatment of liver diseases, and accumulating evidences have revealed that MSCs can alleviate liver fibrosis and improve liver function [38,39,40,41]. Moreover, MSC transplantation has been demonstrated to be effective for the treatment of human liver fibrosis in clinical trials [42]. Nevertheless, the fact of cellular rejection and the risk for iatrogenic tumor formation remain unresolved in MSC transplantation [43]. Thus, MSC-derived exosomes that have no risk for tumor formation and less immunogenicity [15] may be a better choice. A large number of studies have revealed the ameliorative effect of MSCs-derived exosomes on liver fibrosis [17,44,45]. In the present study, exosomes were successfully isolated from human BMSCs and used in the subsequent experiments.

Exosomes have been found to carry many contents, such as miRNAs, to mediate intercellular communication [46]. miR-148a from MSC-derived exosomes prevents liver fibrosis by targeting KLF6/STAT3 pathway [18]. MSC-derived exosomal miR-618 dampens the development of liver fibrosis by targeting Smad4 [33]. Here, the miR-26a expression was reduced in BMSC-derived exosomes. Treatment of miR-26a mimic-Exo decreased the EDU-positive rate and the protein level of α-SMA and collagen I but enhanced the apoptosis rate of LX2 cells. The enhancement of ECM proteins is a major feature of liver fibrosis [1]. HSCs, the main source of ECM proteins, are quiescent under normal physiological state, which can be activated and transdifferentiated upon pathological conditions resulting from liver injury [47]. HSCs, activated by cytokines and fibrogenic regulators, cause the generation of the section of collagens and α-SMA, which contribute to the ECM deposition [47,48,49]. Activated HSCs serve as the myofibroblasts, which impel the fibrotic responses and promote the progression of liver fibrosis [47,50]. Thus, upregulation of BMSC-derived exosomal miR-26a suppressed the HSC activation. Moreover, injection of miR-26a mimic-Exo reduced liver fibrosis, and improved the liver function in CCL4-induced mice. Taken together, MSC-derived exosomal miR-26a relieved liver fibrosis.

Moreover, MSC-derived exosomal miR-26a induced ferroptosis in LX2 cells, as evidenced by the increased level of ROS, Fe^2+^, and MDA and the decreased GHS level. Furthermore, the application of DFO further verified that MSC-derived exosomal miR-26a promoted ferroptosis to relieve liver fibrosis in LX2 cells. More importantly, miR-26a directly bound to SLC7A11 mRNA and negatively regulated the level of SLC7A11 in LX2 cells. SLC7A11 is a critical protein related to the progression of ferroptosis, and it is an ingredient of the cystine/glutamate antiporter to be responsible for providing the cysteine necessary for the synthesis of GSH [7]. SLC7A11 has been demonstrated to have potentially therapeutic effects in preventing or treating liver fibrosis. For instance, TRIM26 induces HSC ferroptosis to inhibit liver fibrosis by the ubiquitinated degradation of SLC7A11 [51]. Sorafenib evokes HSC ferroptosis to attenuate liver fibrosis by regulating the HIF-1α/SLC7A11 axis [52]. Wogonoside dampens liver fibrosis by evoking HSC ferroptosis through the SOCS1/P53/SLC7A11 axis [53]. In the present study, overexpression of SLC7A11 reversed the miR-26a mimic-Exo-induced alterations in the level of ROS, Fe^2+^, MDA, and GSH in LX2 cells, indicating that BMSC-derived exosomal miR-26a elicited ferroptosis of HSCs by regulating SLC7A11.

In summary, our results expounded that BMSC-derived exosomal miR-26a induced ferroptosis to alleviate liver fibrosis by regulating SLC7A11. However, there are still some limitations in this study that needed to be addressed in the future. The direct role of miR-26a/SLC7A11 axis in liver fibrosis can be investigated both *in vitro* and *in vivo*. Whether the treatment of miR-26a will not affect the expression of SLC7A11 mRNA but modulate the post-transcriptionally protein level should be confirmed by RT-qPCR experiment in the following experiment. A SLC7A11 knockdown together with miR-26a treatment can be conducted to further confirm that miR-26a control liver fibrosis through the SLC7A11 pathway. In addition, more preclinical and clinical trials are essential in the subsequent study for the clinical application of exosomes. Collectively, our results provide the pre-clinical evidence for the discovery of the possible target for the treatment of liver fibrosis.

## References

[1] Kisseleva T, Brenner D. Molecular and cellular mechanisms of liver fibrosis and its regression. Nat Rev Gastroenterol Hepatol. 2021;18(3):151–66. 10.1038/s41575-020-00372-7. 33128017

[2] Hernandez-Gea V, Friedman SL. Pathogenesis of liver fibrosis. Annu Rev Pathol. 2011;6:425–56. 10.1146/annurev-pathol-011110-130246. 21073339

[3] Friedman SL, Pinzani M. Hepatic fibrosis 2022: Unmet needs and a blueprint for the future. Hepatology. 2022;75(2):473–88. 10.1002/hep.32285. PMC12179971 34923653

[4] Böttcher K, Pinzani M. Pathophysiology of liver fibrosis and the methodological barriers to the development of anti-fibrogenic agents. Adv Drug Deliv Rev. 2017;121:3–8. 10.1016/j.addr.2017.05.016. 28600202

[5] Jia Y, Wang F, Guo Q, Li M, Wang L, Zhang Z, et al. Curcumol induces RIPK1/RIPK3 complex-dependent necroptosis via JNK1/2-ROS signaling in hepatic stellate cells. Redox Biol. 2018;19:375–87. 10.1016/j.redox.2018.09.007. PMC6142373 30237126

[6] Luo P, Liu D, Zhang Q, Yang F, Wong YK, Xia F, et al. Celastrol induces ferroptosis in activated HSCs to ameliorate hepatic fibrosis via targeting peroxiredoxins and HO-1. Acta Pharm Sin B. 2022;12(5):2300–14. 10.1016/j.apsb.2021.12.007. PMC9136576 35646542

[7] Dixon SJ, Lemberg KM, Lamprecht MR, Skouta R, Zaitsev EM, Gleason CE, et al. Ferroptosis: an iron-dependent form of nonapoptotic cell death. Cell. 2012;149(5):1060–72. 10.1016/j.cell.2012.03.042. PMC3367386 22632970

[8] Stockwell BR, Friedmann Angeli JP, Bayir H, Bush AI, Conrad M, Dixon SJ, et al. Ferroptosis: A regulated cell death nexus linking metabolism, redox biology, and disease. Cell. 2017;171(2):273–85. 10.1016/j.cell.2017.09.021. PMC5685180 28985560

[9] Zhang Z, Guo M, Shen M, Kong D, Zhang F, Shao J, et al. The BRD7-P53-SLC25A28 axis regulates ferroptosis in hepatic stellate cells. Redox Biol. 2020;36:101619. 10.1016/j.redox.2020.101619. PMC7330619 32863216

[10] Zhang Z, Guo M, Li Y, Shen M, Kong D, Shao J, et al. RNA-binding protein ZFP36/TTP protects against ferroptosis by regulating autophagy signaling pathway in hepatic stellate cells. Autophagy. 2020;16(8):1482–505. 10.1080/15548627.2019.1687985. PMC7469536 31679460

[11] Li Y, Jin C, Shen M, Wang Z, Tan S, Chen A, et al. Iron regulatory protein 2 is required for artemether -mediated anti-hepatic fibrosis through ferroptosis pathway. Free Radic Biol Med. 2020;160:845–59. 10.1016/j.freeradbiomed.2020.09.008. 32947011

[12] Tsurusaki S, Tsuchiya Y, Koumura T, Nakasone M, Sakamoto T, Matsuoka M, et al. Hepatic ferroptosis plays an important role as the trigger for initiating inflammation in nonalcoholic steatohepatitis. Cell Death Dis. 2019;10(6):449. 10.1038/s41419-019-1678-y. PMC6579767 31209199

[13] Sun X, Niu X, Chen R, He W, Chen D, Kang R, et al. Metallothionein-1G facilitates sorafenib resistance through inhibition of ferroptosis. Hepatology. 2016;64(2):488–500. 10.1002/hep.28574. PMC4956496 27015352

[14] Zhang Z, Yao Z, Wang L, Ding H, Shao J, Chen A, et al. Activation of ferritinophagy is required for the RNA-binding protein ELAVL1/HuR to regulate ferroptosis in hepatic stellate cells. Autophagy. 2018;14(12):2083–103. 10.1080/15548627.2018.1503146. PMC6984765 30081711

[15] Greening DW, Gopal SK, Xu R, Simpson RJ, Chen W. Exosomes and their roles in immune regulation and cancer. SemCell Dev Biol. 2015;40:72–81. 10.1016/j.semcdb.2015.02.009. 25724562

[16] He C, Zheng S, Luo Y, Wang B. Exosome theranostics: Biology and translational medicine. Theranostics. 2018;8(1):237–55. 10.7150/thno.21945. PMC5743472 29290805

[17] Rong X, Liu J, Yao X, Jiang T, Wang Y, Xie F. Human bone marrow mesenchymal stem cells-derived exosomes alleviate liver fibrosis through the Wnt/β-catenin pathway. Stem Cell Res Ther. 2019;10(1):98. 10.1186/s13287-019-1204-2. PMC6421647 30885249

[18] Tian S, Zhou X, Zhang M, Cui L, Li B, Liu Y, et al. Mesenchymal stem cell-derived exosomes protect against liver fibrosis via delivering miR-148a to target KLF6/STAT3 pathway in macrophages. Stem Cell Res Ther. 2022;13(1):330. 10.1186/s13287-022-03010-y. PMC9297598 35858897

[19] Valadi H, Ekström K, Bossios A, Sjöstrand M, Lee JJ, Lötvall JO. Exosome-mediated transfer of mRNAs and microRNAs is a novel mechanism of genetic exchange between cells. Nat Cell Biol. 2007;9(6):654–9. 10.1038/ncb1596. 17486113

[20] O’Brien J, Hayder H, Zayed Y, Peng C. Overview of MicroRNA biogenesis, mechanisms of actions, and circulation. Front Endocrinol (Lausanne). 2018;9:402. 10.3389/fendo.2018.00402. PMC6085463 30123182

[21] Liu L, Wang P, Wang YS, Zhang YN, Li C, Yang ZY, et al. MiR-130a-3p alleviates liver fibrosis by suppressing HSCs activation and skewing macrophage to Ly6Clo phenotype. Front Immunol. 2021;12:696069. 10.3389/fimmu.2021.696069. PMC8375151 34421906

[22] Zhang W, Wang Q, Feng Y, Chen X, Yang L, Xu M, et al. MicroRNA-26a protects the heart against hypertension-induced myocardial fibrosis. J Am Heart Assoc. 2020;9(18):e017970. 10.1161/jaha.120.017970. PMC7726969 32865120

[23] Chiang MH, Liang CJ, Lin LC, Yang YF, Huang CC, Chen YH, et al. miR-26a attenuates cardiac apoptosis and fibrosis by targeting ataxia-telangiectasia mutated in myocardial infarction. J Cell Physiol. 2020;235(9):6085–102. 10.1002/jcp.29537. 31990056

[24] Zhang A, Wang H, Wang B, Yuan Y, Klein JD, Wang XH. Exogenous miR-26a suppresses muscle wasting and renal fibrosis in obstructive kidney disease. FASEB J. 2019;33(12):13590–601. 10.1096/fj.201900884R. PMC6894078 31593640

[25] Liang H, Xu C, Pan Z, Zhang Y, Xu Z, Chen Y, et al. The antifibrotic effects and mechanisms of microRNA-26a action in idiopathic pulmonary fibrosis. Mol Ther. 2014;22(6):1122–33. 10.1038/mt.2014.42. PMC4048895 24594795

[26] Han W, Fu X, Xie J, Meng Z, Gu Y, Wang X, et al. MiR-26a enhances autophagy to protect against ethanol-induced acute liver injury. J Mol Med (Berl). 2015;93(9):1045–55. 10.1007/s00109-015-1282-2. PMC4577542 25877859

[27] Xu H, Tian Y, Tang D, Zou S, Liu G, Song J, et al. An endoplasmic reticulum stress-MicroRNA-26a feedback circuit in NAFLD. Hepatology. 2021;73(4):1327–45. 10.1002/hep.31428. 32567701

[28] Zhou J, Li Z, Huang Y, Ju W, Wang D, Zhu X, et al. MicroRNA-26a targets the mdm2/p53 loop directly in response to liver regeneration. Int J Mol Med. 2019;44(4):1505–14. 10.3892/ijmm.2019.4282. 31364731

[29] Liu X, Ma H, Wu R, Wang H, Xu H, Li S, et al. Identification of liver fibrosis-related MicroRNAs in human primary hepatic stellate cells using high-throughput sequencing. Genes (Basel). 2022;13(12):2201. 10.3390/genes13122201. PMC9778123 36553468

[30] Zeng Y, Ma W, Ma C, Ren X, Wang Y, Fu Z. USP15 alleviates the cerulein-induced cell apoptosis and inflammatory injury to AR42J cells through regulating TAB2/3/NF-κB pathway in acute pancreatitis. Signa Vitae. 2021;17(5):130–6. 10.22514/sv.2021.142.

[31] Maru VP, Madkaikar M, Sattar S, Chauhan R, Devi RKS. Response of intra canal medicaments on viability and survival of SHEDs. J Clin Pediatr Dent. 2022;46(5):65–71. 10.22514/jocpd.2022.009. 36624916

[32] National Research Council Committee for the Update of the Guide for the C, Use of Laboratory A. The National Academies Collection: Reports funded by National Institutes of Health. Guide for the Care and Use of Laboratory Animals. Washington (DC): National Academies Press (US)Copyright © 2011, National Academy of Sciences; 2011.

[33] Sun C, Shi C, Duan X, Zhang Y, Wang B. Exosomal microRNA-618 derived from mesenchymal stem cells attenuate the progression of hepatic fibrosis by targeting Smad4. Bioengineered. 2022;13(3):5915–27. 10.1080/21655979.2021.2023799. PMC8973762 35199612

[34] Hu LT, Wang BY, Fan YH, He ZY, Zheng WX. Exosomal miR-23b from bone marrow mesenchymal stem cells alleviates oxidative stress and pyroptosis after intracerebral hemorrhage. Neural Regen Res. 2023;18(3):560–7. 10.4103/1673-5374.346551. PMC9727431 36018178

[35] Babaha F, Yazdani R, Shahkarami S, Esfahani ZH, Abolhahassani H, Sadr M, et al. Evaluation of miR-210 expression in common variable immunodeficiency: patients with unsolved genetic defect. Allergol Immunopathol (Madr). 2021;49(2):84–93. 10.15586/aei.v49i2.39. 33641299

[36] Gomez MK, Thomson JP, Grimes GR, Wang AT, Churchman M, O’Connor MJ, et al. Identifying and overcoming a mechanism of resistance to WEE1 kinase inhibitor AZD1775 in high grade serous ovarian cancer cells. Eur J Gynaecol Oncol. 2022;43(2):183–95. 10.31083/j.ejgo4302024.

[37] Lee EH, Lee JN, Park S, Chun SY, Yoon BH, Chung J-W, et al. Inhibition of TRPM7 suppresses migration and invasion of prostate cancer cells via inactivation of ERK1/2, Src and Akt pathway signaling. J Men’s Health. 2022;18(7):1–10. 10.31083/j.jomh1807144.

[38] Eom YW, Shim KY, Baik SK. Mesenchymal stem cell therapy for liver fibrosis. Korean J Intern Med. 2015;30(5):580–9. 10.3904/kjim.2015.30.5.580. PMC4578027 26354051

[39] Liu WH, Song FQ, Ren LN, Guo WQ, Wang T, Feng YX, et al. The multiple functional roles of mesenchymal stem cells in participating in treating liver diseases. J Cell Mol Med. 2015;19(3):511–20. 10.1111/jcmm.12482. PMC4369809 25534251

[40] Alfaifi M, Eom YW, Newsome PN, Baik SK. Mesenchymal stromal cell therapy for liver diseases. J Hepatol. 2018;68(6):1272–85. 10.1016/j.jhep.2018.01.030. 29425678

[41] Duan X, Wang Y, He J, Peng Y, Yu J, Sun Y. Progress of the application of stem cell therapy for end-stage liver disease. Zhong Nan Da Xue Xue Bao Yi Xue Ban. 2017;42(4):457–62. 10.11817/j.issn.1672-7347.2017.04.015. 28490706

[42] Liang J, Zhang H, Zhao C, Wang D, Ma X, Zhao S, et al. Effects of allogeneic mesenchymal stem cell transplantation in the treatment of liver cirrhosis caused by autoimmune diseases. Int J Rheum Dis. 2017;20(9):1219–26. 10.1111/1756-185x.13015. 28217916

[43] Koch M, Lehnhardt A, Hu X, Brunswig-Spickenheier B, Stolk M, Bröcker V, et al. Isogeneic MSC application in a rat model of acute renal allograft rejection modulates immune response but does not prolong allograft survival. Transpl Immunol. 2013;29(1–4):43–50. 10.1016/j.trim.2013.08.004. 23994720

[44] Ellakany AR, El Baz H, Shoheib ZS, Elzallat M, Ashour DS, Yassen NA. Stem cell-derived exosomes as a potential therapy for schistosomal hepatic fibrosis in experimental animals. Pathog Glob Health. 2023;1–21. 10.1080/20477724.2023.2240085. PMC11338202 37519008

[45] Lin Y, Yan M, Bai Z, Xie Y, Ren L, Wei J, et al. Huc-MSC-derived exosomes modified with the targeting peptide of aHSCs for liver fibrosis therapy. J Nanobiotechnol. 2022;20(1):432. 10.1186/s12951-022-01636-x. PMC9526331 36183106

[46] van Niel G, D’Angelo G, Raposo G. Shedding light on the cell biology of extracellular vesicles. Nat Rev Mol Cell Biol. 2018;19(4):213–28. 10.1038/nrm.2017.125. 29339798

[47] Tsuchida T, Friedman SL. Mechanisms of hepatic stellate cell activation. Nat Rev Gastroenterol Hepatol. 2017;14(7):397–411. 10.1038/nrgastro.2017.38. 28487545

[48] Zhang CY, Yuan WG, He P, Lei JH, Wang CX. Liver fibrosis and hepatic stellate cells: Etiology, pathological hallmarks and therapeutic targets. World J Gastroenterol. 2016;22(48):10512–22. 10.3748/wjg.v22.i48.10512. PMC5192262 28082803

[49] Shi J, Zhao J, Zhang X, Cheng Y, Hu J, Li Y, et al. Activated hepatic stellate cells impair NK cell anti-fibrosis capacity through a TGF-β-dependent emperipolesis in HBV cirrhotic patients. Sci Rep. 2017;7:44544. 10.1038/srep44544. PMC5349579 28291251

[50] Higashi T, Friedman SL, Hoshida Y. Hepatic stellate cells as key target in liver fibrosis. Adv Drug Deliv Rev. 2017;121:27–42. 10.1016/j.addr.2017.05.007. PMC5682243 28506744

[51] Zhu Y, Zhang C, Huang M, Lin J, Fan X, Ni T. TRIM26 induces ferroptosis to inhibit hepatic stellate cell activation and mitigate liver fibrosis through mediating SLC7A11 ubiquitination. Front Cell Dev Biol. 2021;9:644901. 10.3389/fcell.2021.644901. PMC8044755 33869196

[52] Yuan S, Wei C, Liu G, Zhang L, Li J, Li L, et al. Sorafenib attenuates liver fibrosis by triggering hepatic stellate cell ferroptosis via HIF-1α/SLC7A11 pathway. Cell Prolif. 2022;55(1):e13158. 10.1111/cpr.13158. PMC8780895 34811833

[53] Liu G, Wei C, Yuan S, Zhang Z, Li J, Zhang L, et al. Wogonoside attenuates liver fibrosis by triggering hepatic stellate cell ferroptosis through SOCS1/P53/SLC7A11 pathway. Phytother Res. 2022;36(11):4230–43. 10.1002/ptr.7558. 35817562

